# Autoimmune adrenal insufficiency in children: a hint for polyglandular syndrome type 2?

**DOI:** 10.1186/s13052-023-01502-y

**Published:** 2023-07-29

**Authors:** Marta Arrigoni, Paolo Cavarzere, Lara Nicolussi Principe, Rossella Gaudino, Franco Antoniazzi

**Affiliations:** 1 Pediatric Division, Department of Pediatrics, University Hospital of Verona, Piazzale Stefani 1, 37126 Verona, Italy; 2 Pediatric Clinic, Department Surgical Sciences, Dentistry, Gynecology and Pediatrics, University of Verona, Verona, Italy; 3 Regional Center for the Diagnosis and Treatment of Children and Adolescents Rare Skeletal Disorders. Pediatric Clinic, Department of Surgical Sciences, Dentistry, Gynecology and Pediatrics, University of Verona, Verona, Italy

**Keywords:** Schmidt’s syndrome, Autoimmune polyglandular syndrome type 2, Primary adrenal insufficiency, Hashimoto’s thyroiditis, Case report

## Abstract

**Background:**

Primary adrenal insufficiency (PAI) in childhood is a life-threatening disease most commonly due to impaired steroidogenesis. Differently from adulthood, autoimmune adrenalitis is a rare condition amongst PAI’s main aetiologies and could present as an isolated disorder or as a component of polyglandular syndromes, particularly type 2. As a matter of fact, autoimmune polyglandular syndrome (APS) type 2 consists of the association between autoimmune Addison’s disease, type 1 diabetes mellitus and/or Hashimoto’s disease.

**Case presentation:**

We report the case of an 8-year-old girl who presented Addison’s disease and autoimmune thyroiditis at an early stage of life. The initial course of the disease was characterized by numerous crises of adrenal insufficiency, subsequently the treatment was adjusted in a tertiary hospital with improvement of disease control.

**Conclusions:**

APS type 2 is a rare condition during childhood, probably because it may remain latent for long periods before resulting in the overt disease. We recommend an early detection of APS type 2 and an adequate treatment of adrenal insufficiency in a tertiary hospital. Moreover, we underline the importance of a regular follow-up in patients with autoimmune diseases, since unrevealed and incomplete forms are frequent, especially in childhood.

## Background

Primary adrenal insufficiency (PAI) is a life-threatening adrenal disease characterised by an inadequate biosynthesis of glucocorticoids, with or without deficiency in mineralocorticoids and variation of adrenal androgens [1]. For what concerns the clinical presentation, PAI’s detection could be challenging due to aspecific and subtle symtpoms (such as muscle weakness, weight loss, abdominal pain, fatigue, anorexia), although rarely the disease’s presentation involves an acute adrenal crisis [1]. PAI involves a vast range of adrenocortical disorders, the most common of which in childhood is represented by congenital adrenal hyperplasia (CAH), in most cases due to 21-hydroxylase deficiency. Other etiologies involve abnormal adrenal development, such as adrenal hypoplasia, resistance to ACTH action and adrenal destruction, and other inherited conditions such as X-linked adrenoleukodystrophy, triple A (Allgrove syndrome) or multisystemic growth restriction syndromes. Beyond inherited diseases, adrenal insufficiency could be the result of acquired illnesses, such as bilateral adrenal haemorrhage, infections, drugs or autoimmune adrenalitis, with a cell-mediated immune mechanism of adrenal cortex’s destruction [1–3]. In this last case, antibodies against steroid 21-hydroxylase account for approximately 90% of cases [1]. In a large nationwide cohort study held in Italy, autoimmune PAI was detected in 16.5% of patients, while inherited conditions were documented in 73.5% of subjects [3]. Actually, differently from adult aetiologies in which the autoimmune destruction represents the predominant cause, autoimmune adrenalitis is a rare disorder in children [4].

Autoimmune primary adrenal insufficiency presents as an isolated disease or as a component of the so-called polyglandular syndromes. Autoimmune polyglandular syndrome (APS) is defined as the association of two or more endocrine disorders, which can arise at different stages during follow-up, remaining latent even for a long time [5]. APS type 1 is a rare recessive disease characterised by the presence of chronic mucocutaneous candidiasis, chronic hypoparathyroidism and Addison’s disease, which usually begin before 5, 10 and 15 years of age, respectively [1]. Its pathogenesis relates to different mutations of the AutoImmune Regulator (AIRE) gene, which plays a key role in the regulation of organ-specific antigen expression in medullary thymic epithelial cells [6, 7]. Differently from APS type 1, APS type 2 has been found strongly associated with the major histocompatibility complex, particularly with class II HLA alleles, and it is characterised by the occurrence of autoimmune Addison’s disease, an essential element to set the diagnosis, and either Hashimoto’s disease or type 1 diabetes mellitus, or both [5, 8]. In addition, a number of other minor manifestations could be associated with this syndrome, such as chronic atrophic gastritis with or without pernicious anaemia, vitiligo, alopecia or gonadal failure [9]. These features could be present at the same time in APS type 1, which is furthermore associated with a number of other diseases that include autoimmune hepatitis, malabsorption, asplenia, pure red cell aplasia and others [10].

## Case presentation

An 8-year-old girl was admitted to the Paediatric Emergency Department (ED) of a suburban hospital with complaints of nausea, persistent vomiting and abdominal pain that had begun a few hours earlier, in the absence of other symptoms. Her past medical history was silent except for a recently appeared alopecia and for a familial predisposition for autoimmune thyroid disease. Particularly, her father is affected by Hashimoto’s thyroiditis while her mother was affected by Grave’s disease and underwent thyroidectomy. They are both taking levothyroxine treatment. Recently the patient’s twin sister developed an autoimmune thyroiditis with subclinical hypothyroidism that has never needed any replacing therapy. She was investigated for other autoimmune diseases, which resulted completely negative. She was born at term after an uneventful twin pregnancy from not-related parents; she had never shown any allergy nor any ongoing infection. In the ED, she appeared in poor general conditions, dehydrated and with a suntanned appearance, while the rest of her physical examination was unremarkable. Her blood analysis showed a severe hypoglycaemia (1.39 mmoL/L), hyponatremia (126 mmoL/L) and hyperkalaemia (6.1 mmoL/L), hence intravenous hydrocortisone and a 5% dextrose perfusion with a sodium chloride supplementation was promptly administered with a stabilisation of the clinical situation. Further blood analysis showed low cortisol (< 0.50 nmoL/L), normal renin (44.9 mU/L, normal values 5.5–80 mU/L), low aldosterone levels (26.9 pmoL/L, normal values 138–831 pmol/L) with high adrenocorticotropic hormone (ACTH > 278 pmoL/L), confirming the suspect of primary adrenal insufficiency. She therefore began a hydrocortisone treatment (15 mg/m^2^/day) and was taken in charge by the paediatric endocrinologists of the peripheral hospital, with little improvements. During the following months, she experienced many relapses of acute adrenal insufficiency that needed several admissions to the ED with clinical evidence of dehydration and dyselectrolytemia with hyponatremia and hyperkalemia. She was therefore brought to the attention of our Paediatric Endocrinology Department, where she underwent further investigations to understand the aetiology of the disease. Following the identification of significantly high renin level (631.2 mU/L, normal values 5.5–80 mU/L), we introduced a mineralocorticoid replacement therapy with fludrocortisone (0.1 mg/day), with a remarkable clinical improvement and without further relapses of symptoms related to acute adrenal insufficiency. Moreover, we identified high levels of 21-hydroxylase antibodies, which led us to the diagnosis of autoimmune Addison disease, while the remaining autoimmunity panels related to diabetes mellitus, thyroid disease, and atrophic gastritis were negative. Nevertheless, a subclinical hypothyroidism was pointed out, with TSH levels (6.64 mU/L) slightly above threshold.

**Fig. 1:**
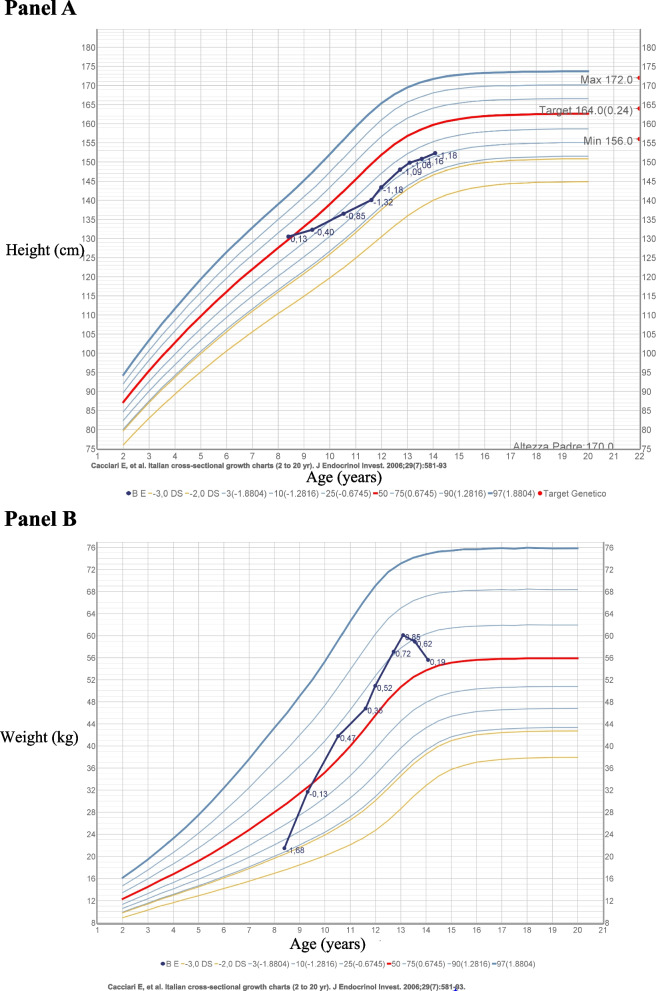
Height and weight growth curves during patient’s follow-up

During the follow-up, the hydrocortisone’s dosage was adjusted according to the patient’s needs, and she underwent constant auxological evaluations, which showed a decrease in height velocity and an increase of weight (Fig. 1).

At the age of 11 we repeated all the autoimmunity panels and we found a positivity for thyroid peroxidase (65 kU/L) and thyroglobulin antibodies (72 UI/mL), with a thyroid ultrasound pattern described as dishomogeneous and pseudo nodular, leading us to the diagnosis of autoimmune thyroiditis. Her thyroid function always remained well-compensated and thus she never needed a substitutive treatment with levothyroxine.

During the subsequent follow-up, she attained menarche in August 2021 at the age of 12 years and 6 months, and, at a two years’ distance from menarche, reached a height of 152.3 cm (-1 standard deviations, [SD]), with an improved trend although still below her target height (163.5 cm; mother’s height 170 cm, father’s height 170 cm). Moreover, she had a considerable weight improvement, her body mass index (BMI) decreased from 95° to 82° centile, measured at the last evaluation in March 2023. Her adrenal function always remained well-compensated (cortisol 445 nmol/L, ACTH 9.89 pmol/L, renin 59.10 mU/L, aldosterone 41.61 pmol/L) and allowed us to progressively decrease the dose of hydrocortisone (up to 14.5 mg/m^2^/day), keeping the fludrocortisone dose stable.

The association between Addison’s disease and Hashimoto’s disease belongs to the so-called Schmidt syndrome or APS type 2, which is the definitive diagnosis for our patient.

## Discussion and conclusions

In the present paper, we describe a case of Schmidt’s syndrome in an Italian 8-year-old girl. Schmidt’s syndrome represents a rare condition during childhood. It must always be considered in the presence of a combination between Addison’s disease and another autoimmune disease, which could show subsequently and not necessarily simultaneously during follow-up. In our case a 3 years’ lapse between the presentation of the first and the second autoimmune disease was testified.

APS type 2 is overall a rare syndrome, with an estimated prevalence around 1.4–4.5 cases per 100.000 inhabitants [4]. Since the syndrome was firstly described in 1981, it has been assumed that it mostly appears after 20 years of age, typically in the third to fourth decades of life, with peaks in middle-aged women and with a prevalence three times higher in females than in the male counterpart [8, 11]. As a matter of fact, Neufeld et al. described how Addison’s disease figures as a component of both APS type 1 and type 2, highlighting its different age of onset among the two syndromes, occurring exclusively in children and young adults in APS type 1 while usually becoming manifest with a peak incidence in midlife in APS type 2 [11].

According to what Betterle et al. reported, the onset of the diseases could be subsequently over the years and overt endocrinopathies could be only the tip of an iceberg [4].

In addition, it has been reported that almost 70% of patients with Addison’s disease develop an autoimmune thyroiditis, while the combination between adrenal insufficiency and type 1 diabetes mellitus occurs in around 52% of the cases [11, 12]. It is noteworthy that, overall, almost 40–50% of patients with Addison’s disease will develop during life at least another autoimmune endocrine disease, hence it is of utmost importance to perform a thorough screening looking for serum autoantibodies (such as thyroid peroxidase, thyroglobulin, glutamate decarboxylase and islet antibodies) and repeating them periodically during the years of follow-up [8, 13].

In our patient, Addison’s disease had its onset at a very early stage of life with a life-threatening presentation and the typical clinical manifestations of acute adrenal insufficiency such as dehydration and dyselectrolytemia.

Data show that approximately 6 to 8% of patients suffering from adrenal insufficiency manifest at least one episode of acute adrenal crisis, especially during coexisting illnesses that tend to precipitate the crisis, which could be even fatal if not adequately treated [14]. Moreover, an Italian multicentric study confirmed that children show a different susceptibility to adrenal crisis, some of them experiencing several episodes. This brings up the need of a specific educational program for caregivers including warnings related to the risk of therapy omission. In particular, it is necessary to educate them to stress dosing with doubled or tripled doses of hydrocortisone per day [14]. Our patient presented multiple relapses of acute adrenal crisis characterized by hyponatremia and hyperkalemia due to mineralocorticoids deficiency that needed several admissions to the ED. These episodes occurred in the first periods of treatment and we believe that two factors contributed to the subsequent reduction: a more adequate and suitable management of the treatment by the caregivers and the introduction of fludrocortisone. In fact, we suggested her parents to contact our Paediatric Endocrinology Service in any case of symptoms possibly related to adrenal crisis. As a matter of fact, we believe that it is of utmost importance to refer children with APS type 2 or other complex endocrinological disorders directly to a specialized reference centre for Paediatric Endocrinology to get the best management of the acute manifestations. Secondarily, the adrenal crisis presented by our patient at the beginning of the treatment were all due to mineralocorticoids deficiency since she always presented normal glycaemia values with low natremia and high kalemia. It is therefore noteworthy to underline the role of mineralocorticoids replacement in patients with autoimmune adrenal insufficiency. As a matter of fact, a model developed by Betterle et al. suggested that an increase in plasma renin concentration represents the first biochemical evidence of adrenal subclinical failure after the appearance of autoantibodies, suggesting that adrenal’s zona glomerulosa may be more prone to autoimmune destruction than zona fasciculata [15, 16]. Nevertheless, the role of renin concentrations in literature seems still quite conflicting [17].

Another aspect related to the lifelong need for steroid replacement therapy in patients with adrenal insufficiency is the necessity of a careful auxological follow-up, since the constant use of supraphysiological doses of hydrocortisone might determine a progressive growth’s slowdown with weight increase, which our girl experienced during her follow-up. Recent guidelines on primary adrenal insufficiency treatment suggest administering hydrocortisone for a total daily dose of 8 mg/m^2^ body surface area, with doses adjusted according to individual needs [18]. However, growth suppression at lower dosages is also possible because dosages above 8 mg/m^2^/day may exceed the physiological range. These suggestions’ aim is to avoid overtreatment, adjusting daily dose depending on clinical status and growth. Excessive weight gain with decreased height velocity or other symptoms or signs of Cushing syndrome indicate excessive glucocorticoid replacement. Measurement of plasma ACTH is typically above the normal range and is not useful for routine monitoring, thus, follow-up of growth and weight velocities and general clinical well-being are most important indicators for dose adjustments. On the contrary, inadequate weight gain, fatigue, anorexia, and hyperpigmentation suggest the need for increased medication dose [18]. Unfortunately, to stabilize our patient’s clinical conditions, especially at the beginning of the treatment, it was necessary to administer supraphysiological dosages, which might have influenced the pubertal growth peak. Nevertheless, the patient did not present the classic stigmata of Cushing’s syndrome. We attempted several times to reduce the hydrocortisone’s dosage, however this tended to worsen the adrenal compensation.

Being aware of the high probability of an association between autoimmune adrenal insufficiency and other endocrinopathies, we performed a thorough follow-up, looking periodically for other autoimmune endocrine diseases. Moreover, at her first admittance in the suburban hospital, our patient reported a recently appeared alopecia, which is one of the minor manifestations of Schmidt syndrome. This management led to the prompt detection of APS type 2, with a final diagnosis set at age 11, which represents a remarkably precocious timing compared to the data available in literature and makes this case noteworthy even for the limited number of children reported with APS type 2. So far, to our knowledge, data available in literature report only 5 children with APS type 2 identified at an age of 11 or younger, but it is interesting to note that the earliest onset occurred in two children of 5 and 8 years old whose syndrome involved a type 1 diabetes [19–23]. Nevertheless, Schmidt’s syndrome with the association between Addison’s disease and autoimmune thyroiditis has never been described in children younger than 11 years old. In order to warrant the best possible management of the diseases with less complications and a better prognosis, it is widely agreed that the main diagnostic goal is to detect APS type 2 at an early stage [8]. To pursue this objective, regular and long-term follow-up must be provided to children with autoimmune diseases. As a matter of fact, we believe that possible reasons for a later detection of APS type 2 could include the high incidence of latent forms before the manifestation of the overt disease and the subsequent evolution of the syndrome amongst the years.

In conclusion, we have described an uncommon presentation of autoimmune APS type 2 with a precocious age of onset, which could contribute to improve awareness concerning APS 2 in children. Therefore, we recommend a prompt referral to a specialized Paediatric Endocrinology Centre and a careful follow-up especially in children with rare and early-onset autoimmune diseases, repeating the tests for other autoimmune diseases over time in order to identify an APS as soon as possible.

## Data Availability

Not applicable.

## Abbreviations

PAI: Primary Adrenal Insufficiency
APS: Autoimmune Polyglandular Syndrome
AIRE: AutoImmune Regulator
CAH: Congenital Adrenal Hyperplasia
ED: Emergency Department
BMI: Body Mass Index
SD: Standard Deviations
